# *Dlc1* deletion activates the canonical Wnt pathway at an early stage and affects subsequent cardiac differentiation in mouse embryonic stem cells

**DOI:** 10.1016/j.gendis.2025.101844

**Published:** 2025-09-09

**Authors:** Wei Wei, Xuechao Jiang, Wenxiang Shi, Junmei Zhou, Rang Xu

**Affiliations:** a Department of Pediatric Cardiology, Xinhua Hospital Affiliated to Shanghai Jiao Tong University School of Medicine, Shanghai 200092, China; b Scientific Research Center, Xinhua Hospital Affiliated to Shanghai Jiao Tong University School of Medicine, Shanghai 200092, China; c Department of Central Laboratory, Shanghai Children's Hospital, Shanghai Jiao Tong University School of Medicine, Shanghai 200062, China

**Keywords:** Cardiac progenitors, Cardiomyocyte differentiation, *Dlc1*, Embryonic stem cell, Wnt signaling

## Abstract

Cardiomyocyte differentiation is an important process during heart development that involves the specification of a series of cardiac lineage cells differentiated from mesendoderm to cardiac mesoderm, precisely regulated by multiple genes and signaling pathways. Our study aims to explore the role of the deleted in liver cancer 1 (*DLC1*) gene in cardiomyocyte differentiation and the molecular mechanisms underlying it. We constructed a *Dlc1* knockout mouse embryonic stem cell (mESC) line using CRISPR/Cas9 and an mESC-cardiomyocyte differentiation model to investigate the role of the *Dlc1* gene in the cardiac progenitor differentiation process. Flow cytometry, real-time PCR, teratoma formation assay, RNA sequencing, and other cellular and molecular biology assays were used to detect the cellular processes and differentiation ability of the knockout cell line at different stages. We found that the cardiac contractility of embryonic bodies was weakened by *Dlc1* deletion at the late stage of differentiation. Moreover, *Dlc1* deficiency promoted mesoderm specification and the subsequent differentiation and accumulation of cardiac progenitors, which may later impair cardiomyocyte generation. RNA sequencing analysis revealed that *Dlc1* deficiency promoted mesoderm specification by activating canonical Wnt signaling, which subsequently enhanced cardiac progenitor differentiation. These results were confirmed by rescue experiments with the Wnt pathway inhibitor XAV-939. Furthermore, we found that the *Dlc1* knockout activated canonical Wnt signaling by affecting the degradation and nuclear transportation of β-catenin. In conclusion, *Dlc1* is involved in regulating embryonic stem cell cardiac differentiation by affecting canonical Wnt signaling at an early stage.

## Introduction

The heart begins beating at the third week of gestation and is the first functioning organ in an embryo. Heart development is highly conserved and follows a similar pattern in different vertebrates.^1^ During this process, the specification and proliferation of myocyte and non-myocyte cell lineages is the key point of heart morphogenesis.^2^ Cardiomyocytes differentiate from cardiac progenitors, which originate from the mesoderm at the gastrulation stage.^1^ Cardiomyocyte lineage specification requires spatial–temporal regulation of multi-signaling pathways at multiple levels, such as the bone morphogenetic protein (BMP), WNT, Notch, and fibroblast growth factor (FGF) signaling pathways, and the precise expression or silence of regulatory and structural genes.^3^^,^^4^ Over the last few decades, knowledge of this regulatory network has grown, yet it remains incompletely understood. Further refinement of this complex regulatory network and identification of new candidate genes may provide a basis for exploring the mechanisms of cardiac diseases, heart regeneration, or remodeling.^5^^,^^6^

Knockout of the protein expression gene deleted in liver cancer 1 (*DLC1*) led to the malformation of multiple organs^7^; therefore, we identified it as a candidate gene for our study on heart development. *DLC1*, located on chromosome 8 (8p21.3–22), is a tumor suppressor gene that is down-regulated or inactivated in various solid tumors.^8^ It is an important member of the DLC family, which includes three Rho GTPase activating proteins (RhoGAP).^9^ The DLC family members (*DLC1*, *DLC2*, and *DLC3)* share a similar structural composition with different subcellular localizations and tissue distribution patterns. Current progress in understanding the biological functions of DLC proteins mainly focuses on their roles in neoplasia. DLC proteins act as a switch to promote the conversion of active RhoGTP to inactive RhoGDP, affecting cytoskeleton formation, cell cycle progression, differentiation, proliferation, adhesion, and migration.^9^, ^10^, ^11^, ^12^ Although *DLC1* is a RhoGAP, RhoGAP-independent pathways via a steroidogenic acute regulatory-related lipid-transfer (START) domain or serine-rich (SR) region have also been identified.^13^^,^^14^

The *Dlc1* gene has been reported to be involved in embryonic development. *In situ* hybridization showed that *Dlc1* transcripts were widely expressed in 10.5 dpc (days post coitum) mouse embryos, such as in the branchial arches, heart, and liver primordia, while its expression was relatively low in the neuroepithelium of the brain and neural tube.^7^ Notably, the role of *Dlc1* in early embryonic development, particularly in cardiovascular system formation, has been demonstrated in animal models. Mohammad and Sabbir et al found that *Dlc1* knockout mice cannot survive beyond 10.5 dpc.^7^^,^^15^ Histological analysis of these 10.5 dpc embryos revealed various degrees of growth retardation in multiple organs, including the hindbrain, branchial arches, heart, and placental vasculature.^7^ The cardiac phenotype showed distorted heart chambers and abnormally enlarged atria and ventricles.^15^

The animal models have sparked our interest in investigating the role *Dlc1* plays in cardiovascular system development. Previously, Shih et al established an endothelial-specific *Dlc1* knockout mouse model, and they found that *Dlc1* played a positive regulatory role in angiogenesis by independently regulating endothelial cell migration through RhoA and paxillin.^16^ However, the cardiac phenotype could not be reproduced in this animal model. Therefore, to elucidate the role of *DLC1* in cardiac development, we investigated the role of *Dlc1* in cardiac progenitor differentiation by constructing a *Dlc1* knockout mESC (mouse embryonic stem cell) monoclonal cell line using CRISPR/Cas9 to mimic cardiomyocyte differentiation. We observed that *Dlc1* deficiency promoted the differentiation and accumulation of the cardiac progenitor, which may impair subsequent cardiomyocyte generation. RNA sequencing analysis further confirmed that *Dlc1* deficiency promoted mesoderm specification by activating canonical Wnt signaling, enhancing cardiac progenitor differentiation.

## Material and methods

### Tissue collection and immunohistochemistry

Mouse embryos of the C57BL/6J genetic background were collected at embryonic day (E) 12.5. Embryos were fixed in 4% paraformaldehyde overnight, embedded in paraffin, and sectioned at a thickness of 4 μm. Anti-Dlc1 antibody (Abcam, ab180697, 1:200) was used for immunohistochemistry.

### mESC culture and differentiation

E14 mESC was given by Prof. Yun Huang from the University of Texas (USA). Mouse embryonic fibroblasts treated with mitomycin C were used as feeder cells for mESC. The culture medium for maintenance includes Knockout DMEM Medium (Gibco), 15% fetal bovine serum (Wisent), 1% non-essential amino acids (Gibco), 1% penicillin-streptomycin (Gibco), 0.1 mM 2-mercaptoethanol (Sigma), and 500 U/mL leukemia inhibitory factor (Millipore).

The hanging drop differentiation process was conducted according to the previous report.^17^ During differentiation, mESCs were cultured in medium without leukemia inhibitory factor. The droplets containing 800 mESCs were cultured on the inner surface of the petri dish lid for 2 days to form embryonic bodies (EBs). Then, the EBs were collected and cultured in non-adhesive dishes for suspension culture for 3 days. At day 6, EBs were collected and cultured in cell culture 96-well plates treated with gelatin for adherent culture for 23 additional days.

To measure the EB size during the 5-day hanging drop culture, EBs were collected and washed with phosphate-buffered saline (PBS), and then transferred into a petri dish for imaging using a stereo microscope (Olympus). ImageJ was used to quantify EB sizes. The beating focal was counted under an inverted phase contrast microscope (Olympus). For rescue experiments, EBs were collected at day 2 and washed with PBS. EBs were then cultured in differentiation medium including 0.5 μM Wnt pathway inhibitor XAV-939 (which promotes β-catenin degradation by inhibiting tankyrase 1 and 2, stabilizing axin in the destruction complex) for 24 h. After that, EBs were washed with PBS 3 times and changed into normal differentiation medium.

### *Dlc1* knockout mESC generation

*Dlc1-*knockout mESC monoclonal cell line was generated by the CRISPR/Cas9 method described previously.^18^ To construct *Dlc1-*gRNA, we added restriction enzyme sites to both ends of the gRNA primer sequences, which were designed with the addition of 5′-CACC-3′ at the 5′ end of the forward primer and 5′-AAAC-3′ at the 5′ end of the reverse primer (Table S1). Due to the preference of the U6 RNA polymerase III promoter in the PX459 vector using a guanine (G) nucleotide as the first base of transcription, an additional G was added at the 5′ end of the forward primer when the gRNA did not begin with G. Correspondingly, a complementary cytosine (C) was added to the reverse primer. Three PX459-*Dlc1-*gRNA plasmids were generated and transfected simultaneously into mESC via the Neon transfection system (Invitrogen). Monoclonal cell lines were sorted into 96-well plates in individual colonies for expanded culture. DNA was extracted and used as a template for genotyping. Two parts of the gRNA-targeted regions were amplified by PCR, and PCR products were connected to pDM19-T vectors for sequencing (Table S2). The candidate *Dlc1-*knockout mESCs were then used for expanded culture, and their protein was extracted for validation.

### Real-time PCR

Wild-type and *Dlc1-*knockout mESC were harvested at a certain time point during the differentiation process. We extracted total RNA by TRIzol reagent (Invitrogen) and performed reverse transcription using Prime Script RT Master Mix (Takara). Real-time PCR was performed on Applied Biosystems QuantStudio3 Real-Time PCR System (Applied Biosystems) using SYBR Premix Ex Taq (Takara). GAPDH was used as an internal control to normalize the expression levels. Primer sequences are shown in Table S2.

### Western blotting

Wild-type and *Dlc1-*knockout mESC were harvested at a certain time point during the differentiation process. Total protein was extracted using RIPA lysis buffer (Beyotime) with phenylmethanesulfonyl fluoride (Beyotime). Nucleus and cytoplasm protein was extracted using a nuclear and cytoplasmic protein extraction kit. The proteins were separated using 6% or 10% SDS-PAGE and then transferred to PVDF membranes (Immobilon). Primary monoclonal antibodies anti-Dlc1 (Abcam, ab180697, 1:1000), anti-β-catenin (Beyotime, AC106, 1:1000), anti-phospho-β-catenin (Cell Signaling Technology, 9561, 1:1000), and anti-non-phospho-β-catenin (Cell Signaling Technology, 8814, 1:1000) were used for Western blotting analysis, and anti-lamin B1 (Beyotime, AF1408, 1:2000), anti-GAPDH (Beyotime, AF0006, 1:1000), and β-actin (Sigma, A2228, 1:5000) were chosen as internal controls. Chemiluminescent immunodetection was performed according to the manufacturer's instructions (Millipore). Pictures were analyzed by the ImageJ software.

### Flow cytometry analysis

EBs were washed and resuspended in PBS. EBs for proliferation analysis were prepared using BeyoClick EdU Cell Proliferation Kit with Alexa Fluor 647 (Beyotime) according to the manufacturer's instructions. Cells were then analyzed by a cell cytometer (BD Bioscience). Data were analyzed by the FlowJo software (BD Bioscience).

### RNA sequencing

Total RNA was extracted from four biological replicates at a certain time point during the differentiation process using the RNeasy Micro Kit. Libraries were constructed using the Stranded mRNA Library Prep Kit and sequenced on Illumina NovaSeq 6000 with an output of more than 40 M reads per sample. The mapping of reads was performed using Hisat2 (version 2.0.4), applying the murine genome (GRCm38.p4 (mm10)). Stringtie (version 1.3.0) was used to count the fragments after genome mapping. To measure gene expression, the FPKM (fragments per kilobase of exon model per million mapped reads) was calculated after data normalization processing. The *P*-value correction for multiple testing was performed using the false discovery rate, and fold-change was calculated according to FPKM. Differentially expressed genes with a false discovery rate lower than 0.05 and positive or negative logged (base 2) fold changes larger than 2 or smaller than −2 were selected. To perform functional enrichment analysis for Gene Ontology (GO) and Kyoto Encyclopedia of Genes and Genomes (KEGG) categories, analysis of differentially expressed genes was conducted. Gene Set Enrichment Analysis (GSEA) was performed using an online tool (https://www.omicshare.com/tools) based on GO and Gene Transcription Regulation Database (GTRD) enrichment analysis.

### Teratoma formation

mESCs were cultured as we described previously. The cells were digested and washed with Dulbecco's PBS. After cell counting, every 1 × 10^6^ wild-type or *Dlc1*^*−/−*^ mESC was resuspended in 100 μL Matrigel (Corning). Every 100 μL mixture was subcutaneously injected into the flank of SCID-Beige mice. After one month, the mice were sacrificed to collect the teratomas. Part of the teratomas were prepared for fixation in 4% paraformaldehyde, embedding, and sectioning, and then applied for hematoxylin-eosin staining. Another part of the teratomas was used for RNA extraction.

### Dual-luciferase reporter assay

To examine how *Dlc1* affected the transcriptional activity of β-catenin, HEK 293T cells were co-transfected with 225 ng wild-type pcDNA3.1-*Dlc1* overexpression plasmids, 130 ng pcDNA3.1-β-catenin plasmids, 130/130 ng TOP/FOP luciferase reporter vector, and 10 ng pRL-TK (Promega). The luciferase activity was measured by the Dual-Glo Luciferase Assay System Kit (Promega).

### Immunofluorescence assay

EBs of a certain time point during the differentiation process were washed and resuspended in PBS. They were fixed with 4% polyformaldehyde followed by 0.2% Triton X-100 treatment for permeabilization, and then incubated with anti-β-catenin antibody (Beyotime, AC106, 1:200). Subsequently, the cells were incubated with Cy3 anti-rabbit secondary antibody (Abcam, ab6939, 1:200). DAPI was used as a nuclear dye. Staining was visualized using a laser scanning confocal microscope (Leica). Pictures were analyzed by the ImageJ software.

### Statistical analysis

Statistical analysis was performed using GraphPad Prism 10 software. Data were presented as mean ± standard deviation from at least three independent replicates of each assay. Statistical significance was evaluated using an unpaired *t*-test or one-way ANOVA, with *p*-values < 0.05 considered statistically significant.

## Results

### Dynamic expression pattern of *DLC1* during mESC-to-cardiomyocyte differentiation

**Figure 1:**
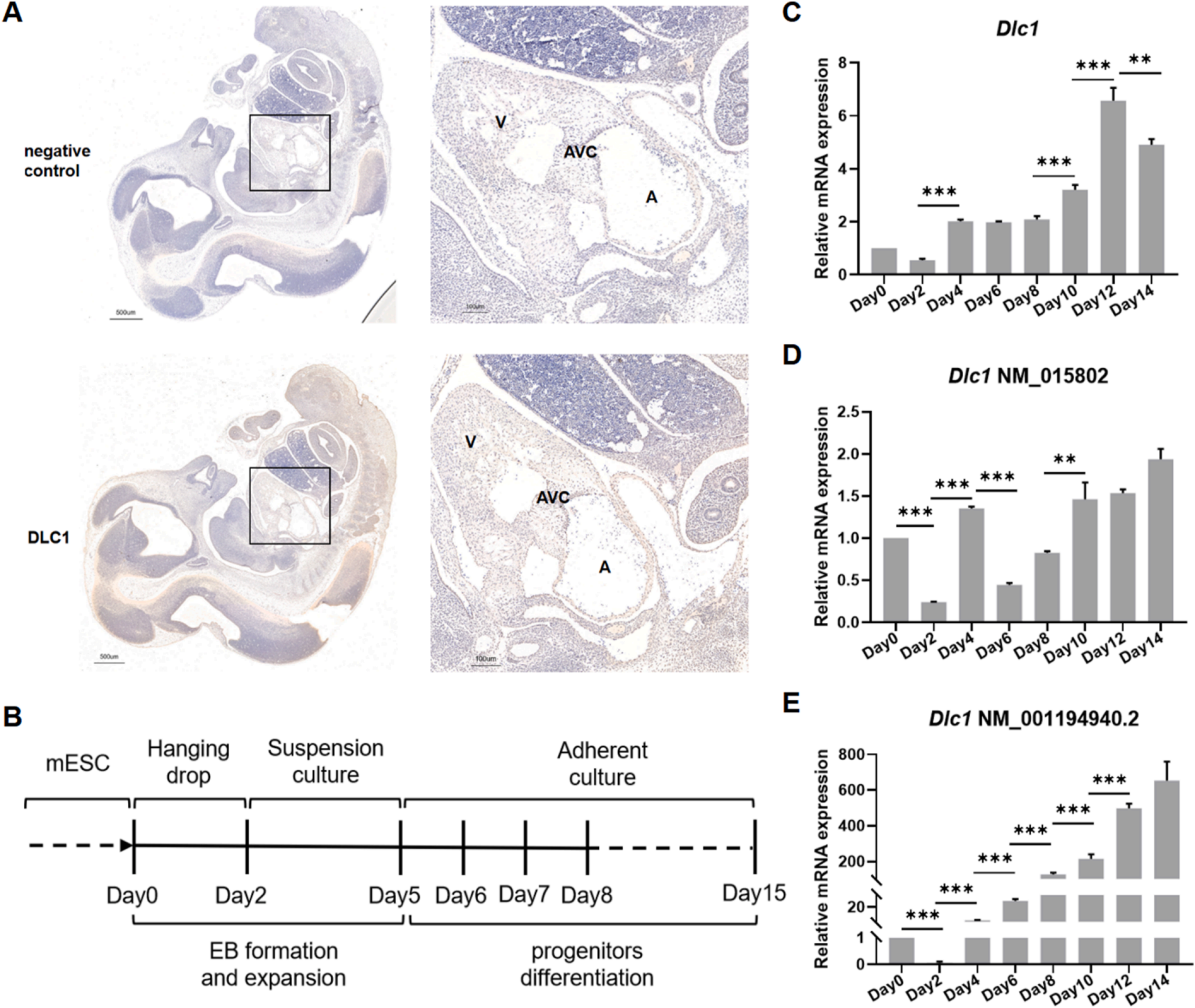
Dynamic Dlc1 expression changes during cardiac progenitor differentiation of mESCs. **(A)** Immunohistochemistry of Dlc1 in E12.5 mouse embryos. AVC, atrio-ventricular canal; A, atrium; V, ventricle. Scale bar, 500/100 μm. **(B)** Schematic illustration of the *in vitro* hanging drop differentiation process. **(C**–**E)** Real-time PCR analysis of common *Dlc1* and two transcript (NM_015802 and NM_001194940.2) expression during mESC differentiation. *n* = 3. Data were shown as mean ± standard deviation; ∗∗*P* < 0.01 and ∗∗∗*P* < 0.001.

To identify candidate genes associated with heart development, we reviewed previous studies and found that *Dlc1* deletion in embryonic mice was lethal and affected the cardiac phenotype, as reported by Mohammad G Sabbir et al,^15^ but the mechanisms are not well understood. We referred to gene expression profiling datasets of human and mouse embryonic hearts to further confirm their underlying relationship with cardiac development. In the human embryonic heart, *DLC1* expression was up-regulated at Carnegie stage 11 and maintained thereafter (Fig. S1A). This expression pattern is similar to that of E10.5 to E18.5 mouse embryonic hearts, according to published datasets (GSE1479) (Fig. S1B). We also performed immunohistochemistry staining on E12.5 mouse embryos and found that *Dlc1* was widely expressed in the embryos, including the mesenchyme of somites, branchial arch, and heart (Fig. 1A). In the heart, *Dlc1* was mainly expressed in the myocardium and endocardium.

While our results and previous studies have demonstrated that *Dlc1* is expressed in the heart during embryonic development, its role in the specification of cardiac lineage is yet to be characterized.^7^ We used a hanging drop method for *in vitro* mESC-to-cardiomyocyte differentiation to investigate the role of *Dlc1* during the process (Fig. 1B). The differentiation of mESC into cardiomyocytes involves a multi-step cell fate transition, including the mesendoderm (day 1–3), cardiac mesoderm (day 4–5), and cardiomyocyte (day 6–15). We observed that total *Dlc1* decreased within the first 2 days, and then gradually increased since day 4 during differentiation (Fig. 1C). The expression patterns of the widely expressed transcript (NM_015802) and heart-specific transcript (NM_001194940.2) were roughly similar to that of total *Dlc1*; however, the heart-specific transcript exhibited more drastic changes (Fig. 1D and E). *Dlc1* was down-regulated during the specification of the gastrulating mesoderm, and it was up-regulated when cardiac progenitors began to differentiate. We hypothesized that *Dlc1* was involved in the differentiation of cardiac progenitor cells.

### *Dlc1* knockout affects mESC cardiomyocyte differentiation

To investigate the role of *Dlc1* in cardiac progenitor differentiation, we used the CRISPR/Cas9 gene editing technique to construct three different *Dlc1-*knockout Nkx2.5-emGFP mESC cell lines that can label cardiac progenitors and chose cell line 2 for further investigation (Fig. S2; Table S3). We found that *Dlc1* deletion did not affect the proliferation and apoptosis of mESC (Fig. S3A and S3B). Meanwhile, the expression of mESC pluripotency-related genes did not change (Fig. S3C–S3E).

**Figure 2:**
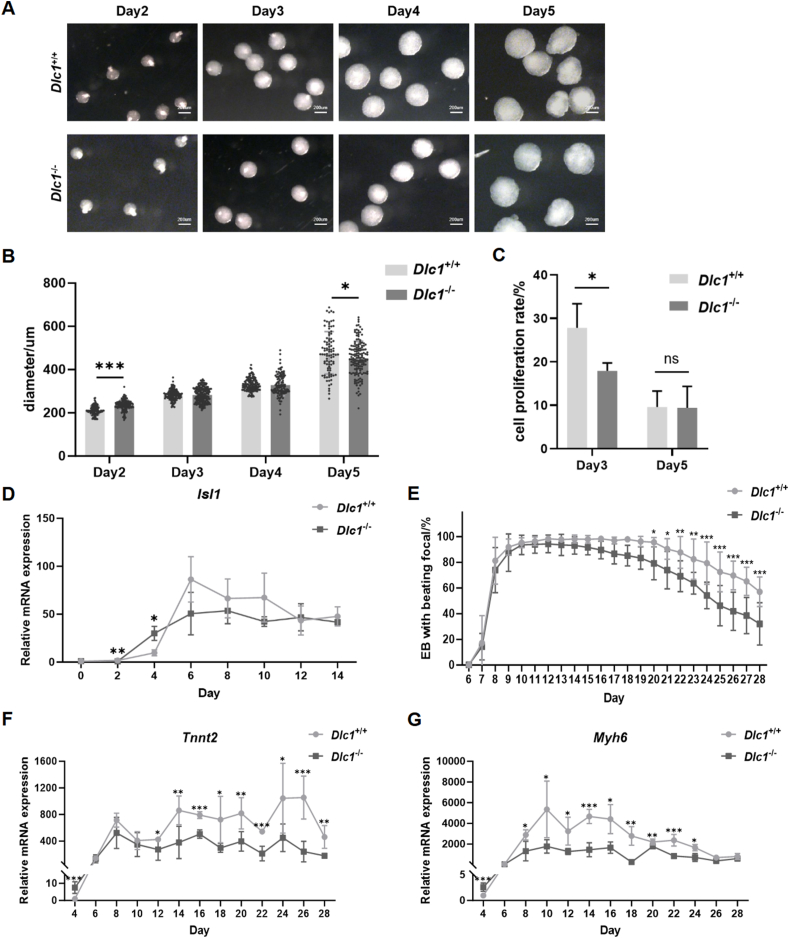
*Dlc1* knockout affects the efficiency of cardiac progenitor differentiation. **(A)** Representative images of embryonic bodies (EBs) during differentiation. Scale bar, 200 μm. **(B)** Quantification of EB size. *n* > 90. **(C)** The proliferation rate of wild-type and *Dlc1*-knockout EBs at day 3 and day 5 detected by flow cytometry. *n* = 3. **(D, F, G)** Real-time PCR analysis of *Isl1*, *Tnnt2*, and *Myh6* expression during mESC differentiation. *n* ≥ 3. **(E)** Quantification of the percentage of beating focal in wild-type and *Dlc1*-knockout EBs during differentiation. Data were shown as mean ± standard deviation; ∗*P* < 0.05, ∗∗*P* < 0.01, and ∗∗∗*P* < 0.001; ns, not significant.

During the first 5 days of the hanging drop process, we observed an increase in EB size in *Dlc1*-knockout mESC compared with the wild-type at day 2 and a decrease at day 5 (Fig. 2A and B). Consistent with changes in EB size, the proliferation of *Dlc1*-knockout EBs decreased at day 3 but did not change at day 5 (Fig. 2C). To monitor cardiac progenitor differentiation efficiency, we measured the Nkx2.5 expression level by flow cytometry. In the control group, Nkx2.5 expression increased rapidly since day 4, peaked at day 6–8, and gradually decreased thereafter (Fig. S4A). We observed a similar expression pattern in *Dlc1*-knockout EBs, which was confirmed by real-time PCR (Fig. S4B). We also measured the expression of islet-1 (*Isl1*), an early-expressed cardiac progenitor marker, and found an increase in *Dlc1*-knockout EBs at day 4 (Fig. 2D). These results indicate that *Dlc1* deficiency favored *Isl1*^+^ cardiac progenitor differentiation at an early stage.

We then tried to access the cardiomyocyte differentiation level. We calculated the percentage of EBs by beating focal after adhesive culture from day 6. *Dlc1*-knockout and wild-type EBs exhibited a similar beating capability, with more than 90% of EBs beating from day 9, indicating successful cardiomyocyte differentiation (Fig. 2E). However, from day 20 onward, the percentage of contracting EBs derived from *Dlc1*^*−/−*^ mESCs progressively declined. Although wild-type EBs also demonstrated a modest reduction in contractility during late stages, the *Dlc1*-knockout line exhibited a significantly more rapid deterioration in contractile maintenance (Fig. 2E). Moreover, cardiomyocyte markers, troponin T2 (*Tnnt2*) and myosin heavy chain 6 (*Myh6*), were down-regulated in *Dlc1*-knockout EBs starting day 8 of differentiation (Fig. 2F and G).

We repeated the key experiments, including the beating focal calculation and verification of marker expression by flow cytometry and real-time PCR in the other two *Dlc1*^*−/−*^ cell lines. The weakened cardiac contractility of *Dlc1*^*−/−*^ EBs was verified in the other two cell lines (Fig. S5A). *Isl1* expression was up-regulated in *Dlc1*^*−/−*^ EBs at day 4, while the expression of *Nkx2.5* remained unaltered (Fig. S5B, S5D–S5G). Moreover, the up-regulation of mesoderm posterior protein 1 (*Mesp1*) (Fig. S5C) and down-regulation of *Tnnt2* and *Myh6* were also confirmed in the other two knockout cell lines (Fig. S5H–S5K). Therefore, *Dlc1* deficiency possibly promotes the differentiation and accumulation of the cardiac progenitor, which may disturb subsequent cardiomyocyte lineage specification.

### RNA-sequencing analysis reveals abnormal embryonic germinal layer differentiation in *Dlc1*-knockout mESC

**Figure 3:**
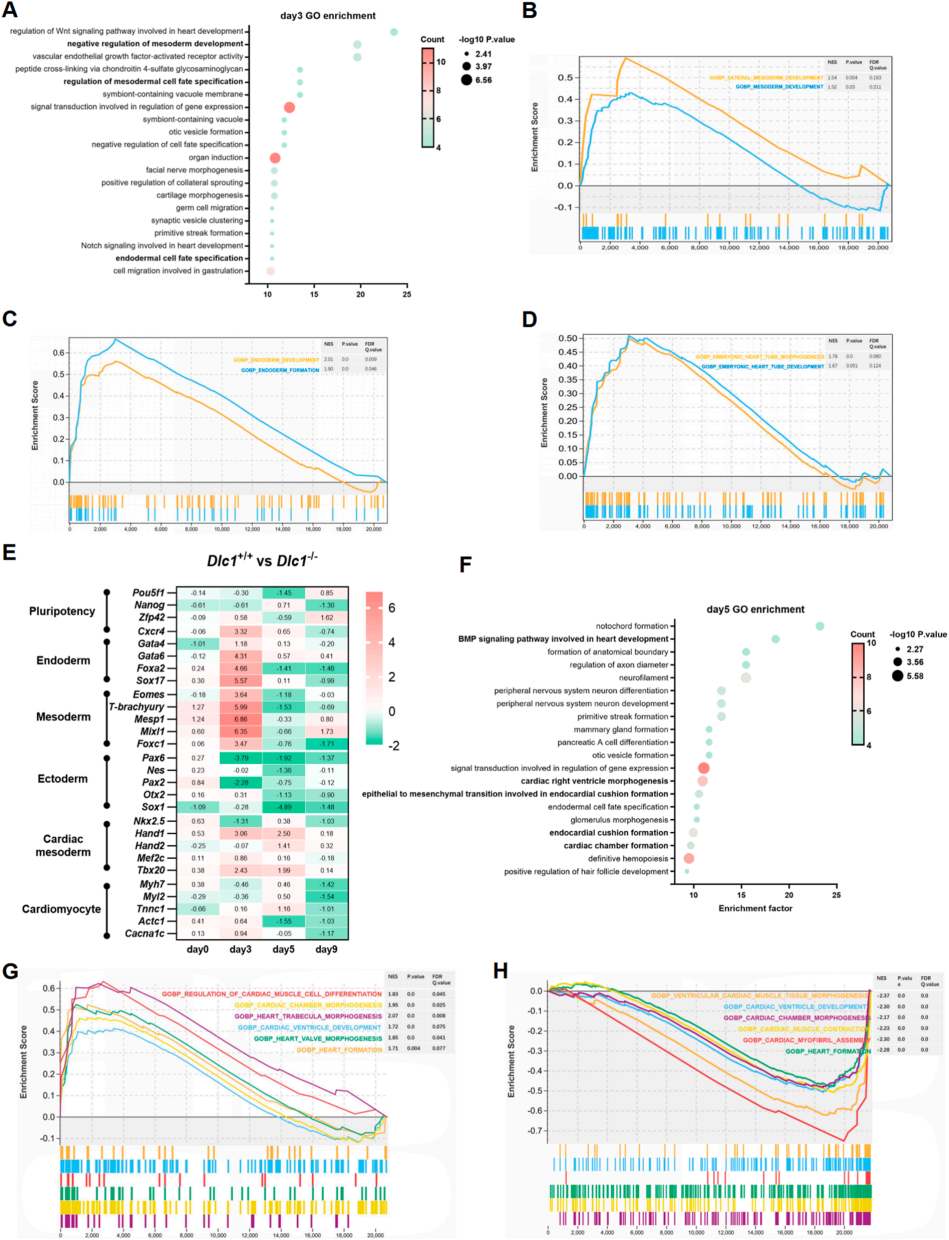
*Dlc1* knockout affects mesoderm-cardiac progenitor differentiation. **(A, F)** Gene Ontology (GO) analysis highlights pathways at day 3 and day 5. **(B**–**D)** Gene Set Enrichment Analysis (GSEA) revealed the differential genes related to indicating GO terms at day 3. NES, normalized enrichment score. **(E)** The heatmap represents up-regulated and down-regulated genes in the control embryonic bodies (EBs) versus *Dlc1*-knockout EBs at day 0, day 3, day 5, and day 9. **(G)** GSEA revealed the differential genes related to indicating GO terms at day 5. **(H)** GSEA revealed the differential genes related to indicating GO terms at day 9.

To further explore the molecular features of *Dlc1*'s involvement in cardiac progenitor differentiation, we performed RNA-sequencing analysis for day 3, day 5, and day 9 on *Dlc1*-knockout and wild-type mESC differentiating to cardiomyocytes. Our results showed that *Dlc1* knockout led to 1006 differentially expressed genes at day 3. During this stage, the gastrulating mesodermal cells differentiate into precardiac progenitors.^1^ We performed GO analysis and found that several of the top 20 pathways were associated with mesoderm or endoderm cell fate specification and heart development (Fig. 3A). GSEA showed that these biological processes were mainly up-regulated in *Dlc1*-knockout EBs (Fig. 3B–D). Among the top up-regulated genes of *Dlc1*-knockout EBs at day 3, we identified some key mesoderm and endoderm markers, such as *Mesp1*, Mix-like homebox 1 (*Mixl1*), Brachyury (*T*), forkhead box A2 (*Foxa2*), SRY-box transcription factor 17 (*Sox17*), and GATA-binding protein 6 (*Gata6*) (Fig. 3E; Fig. S6A and S6B). Notably, certain ectoderm markers, such as paired box 6 (*Pax6*), were significantly down-regulated at this time point (Fig. 3E). We found that *Dlc1* deficiency could promote mesodermal or endodermal cell specification.

Differentially expressed genes were mainly enriched in heart formation-related pathways at day 5, during which the cardiac mesoderm is formed, and progenitors start differentiating towards the cardiac lineages (Fig. 3F).^19^ GSEA showed that the up-regulated genes were mainly associated with biological processes, such as cardiac muscle cell differentiation and heart morphogenesis, in *Dlc1*-knockout EBs (Fig. 3G). Furthermore, some cardiac progenitor markers, such as *Bmp4*, heart and neural crest derivatives expressed 1 (*Hand1*), T-box transcription factor 2 (*Tbx2*), SWI/SNF-related matrix-associated actin-dependent regulator of chromatin subfamily D member 3 (*Smarcd3*), and *Hand2*, were up-regulated in *Dlc1*-knockout EBs at day 5 (Fig. 3E; Fig. S6C). However, during mature myocardial cell differentiation, GSEA showed that heart morphogenesis-related pathways were enriched but down-regulated (Fig. 3H). Several cardiomyocyte markers, such as myosin light chain-2 (*Myl2*), myosin heavy chain-7 (*Myh7*), troponin C1 (*Tnnc1*), and calcium voltage-gated channel subunit alpha 1C (*Cacna1c*), were down-regulated at day 9 (Fig. 3E; Fig. S6D). In summary, *Dlc1* deficiency promotes mesodermal lineage specification, which then contributes to cardiac progenitor accumulation but disturbs further differentiation into cardiomyocytes.

**Figure 4:**
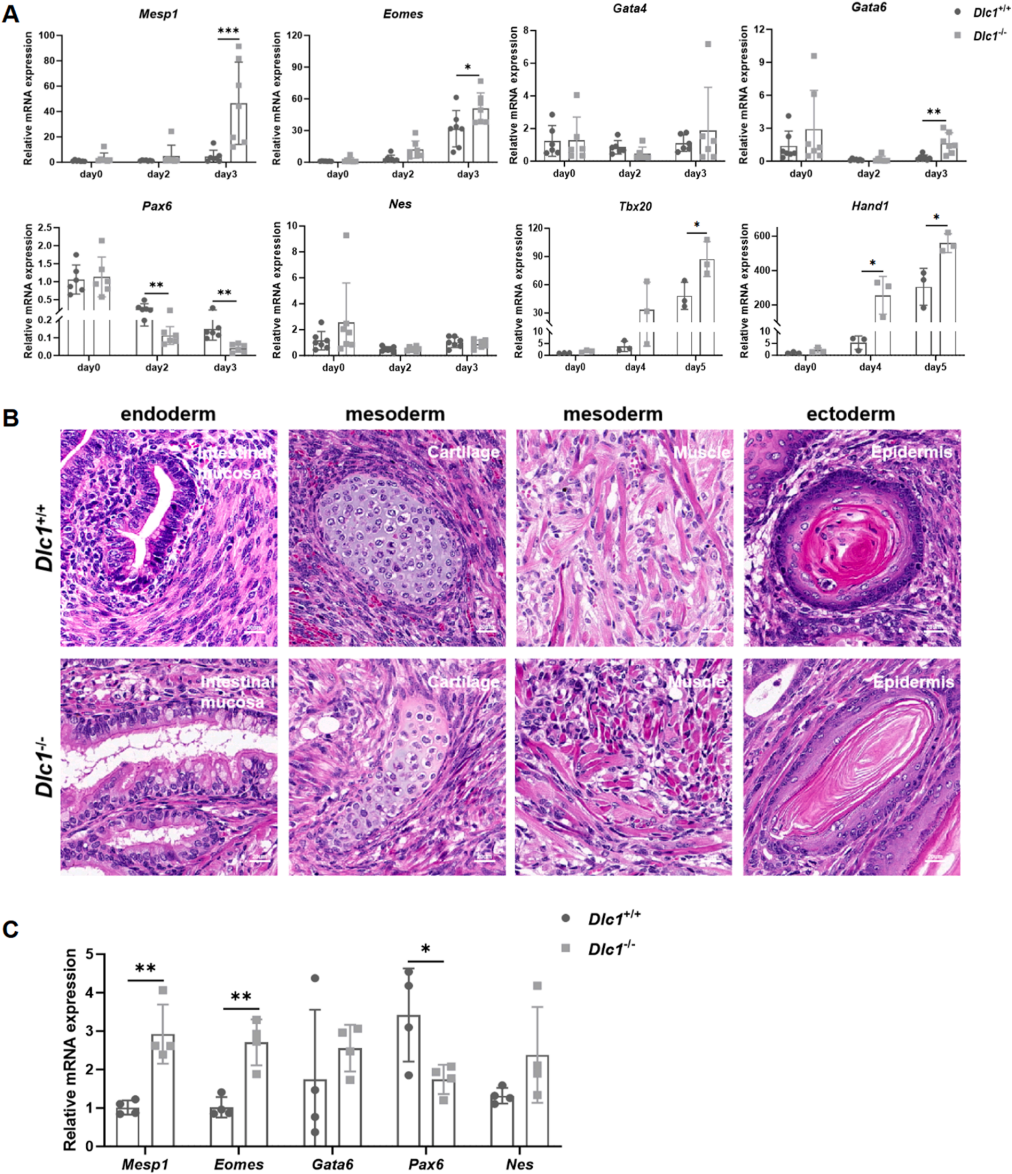
*Dlc1* knockout affects germinal layer differentiation. **(A)** Real-time PCR analysis of germinal layer marker expression at the first three days of mESC differentiation (*n* ≥ 6) and real-time PCR analysis of cardiac progenitor marker expression at day 0, day 4, and day 5 of mESC differentiation (*n* = 3). **(B)** Representative hematoxylin-eosin staining images of teratoma formed by *Dlc1*-knockout and wild-type mESC. Scale bar, 20 μm. **(C)** Real-time PCR analysis of germinal layer marker expression for teratoma. *n* = 4. Data were shown as mean ± standard deviation; ∗*P* < 0.05 and ∗∗*P* < 0.01.

RNA-sequencing results were further substantiated by real-time PCR. Compared with the control group, expression of the mesendoderm markers (*Eomes*, *Mesp1*, and *Gata6*) increased while that of the ectoderm marker (*Pax6*) decreased at day 3, and the expression of cardiac mesoderm markers (*Tbx20* and *Hand1*) increased at day 4 and day 5 in *Dlc1*-knockout EBs (Fig. 4A). Meanwhile, the expression of *Nestin* (*Nes*) remained unchanged at these time points, consistent with the RNA-sequencing results. Teratoma formed by *Dlc1*-knockout and wild-type mESC included tissues originating from the endoderm (intestinal mucosa), mesoderm (cartilage and muscle), and ectoderm (epidermis) (Fig. 4B). Expression levels of mesoderm markers increased in *Dlc1*-knockout teratomas (Fig. 4C). *Dlc1*-knockout mESC seem to have the capability to form three germinal layers; however, they promote mesoderm formation and inhibit ectoderm formation, confirming the RNA-sequencing analysis.

### *Dlc1* regulates Wnt signaling during cardiac progenitor specification

**Figure 5:**
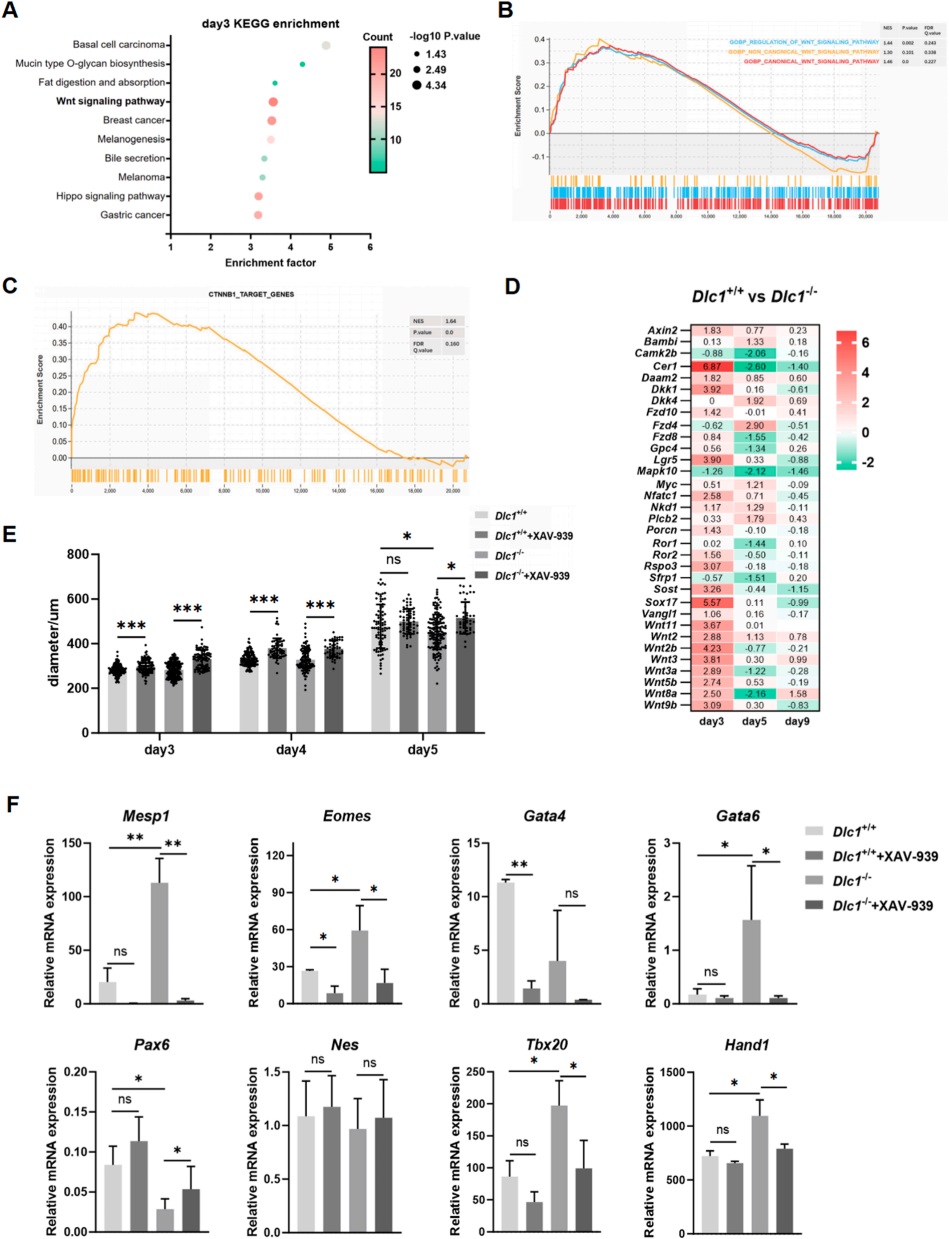
*Dlc1* knockout affects cardiac progenitor differentiation via the Wnt signaling pathway. **(A)** KEGG analysis highlights pathways at day 3. **(B, C)** Gene Set Enrichment Analysis (GSEA) revealed the differentially expressed genes related to Wnt signaling pathways and Ctnnb1 target genes at day 3. NES, normalized enrichment score. **(D)** The heatmap represents genes related to the Wnt signaling pathway in the control embryonic bodies (EBs) versus *Dlc1*-knockout EBs at day 3, day 5, and day 9. **(E)** Quantification of EB size under indicating treatment. *n* > 90. **(F)** Real-time PCR analysis of primary germ layer marker expression at day 3 and cardiac mesoderm marker expression at day 5 under indicating treatment. *n* = 3. Data were shown as mean ± standard deviation; ∗*P* < 0.05 and ∗∗*P* < 0.01; ns, not significant.

To investigate how *Dlc1* deletion was involved in cardiac progenitor differentiation, we queried the RNA-sequencing datasets again. GO and KEGG analyses revealed that Wnt signaling was enriched among the top 10 pathways at day 3 but not at day 5 and day 9. This suggests that *Dlc1* deletion could affect Wnt signaling at an early stage of cardiac lineage specification (Fig. 5A; Fig. S7A and S7B). Wnt is an essential signaling pathway for cardiogenesis, and activating the canonical Wnt pathway has been reported to promote mesoderm specification; however, its negative regulation is necessary for subsequent differentiation of the cardiac progenitors and myocardial cells.^20^ Moreover, the top 10 enriched pathways in KEGG were mainly regarding cardiomyopathy and cardiac muscle contraction at day 9.

GSEA showed that Wnt pathways, especially the canonical Wnt pathway, were significantly up-regulated in *Dlc1*-knockout EBs at day 3 (Fig. 5B). GSEA also demonstrated that differentially expressed genes were mainly enriched in the β-catenin (Ctnnt1) target genes via the GTRD (Fig. 5C). This is consistent with the finding that *Dlc1* deficiency could promote mesodermal lineage specification, indicating *Dlc1* involvement in germinal layer differentiation via the canonical Wnt pathway. At day 5 and day 9, the Wnt pathway was down-regulated according to the GSEA; however, the enrichment was not as significant as that of day 3 (Fig. S7C and S7D). Certain canonical Wnt pathway-related genes, such as the secreted Wnt ligands (*Wnt3*, *Wnt3a*), Fzd receptors, and negative feedback loop regulators (*Cer1*, *Dkk1*), were up-regulated in *Dlc1*-knockout EBs at day 3, whereas no significant differences or even down-regulation were observed at day 5 (Fig. 5D). The down-regulation of Wnt signaling from day 5 aligns with the biphasic regulation required for cardiac progenitor differentiation.

To determine whether *Dlc1* deletion-induced mesoderm specification was achieved through activating the canonical Wnt pathway, we performed a rescue experiment using the Wnt pathway inhibitor XAV-939. *Dlc1*-knockout and wild-type EBs were treated with XAV-939/DMSO at day 2 for 24 h. We observed an increase in the size of inhibitor-treated *Dlc1*-knockout EBs compared with the DMSO-treated group, as well as a restoration of proliferation capability at day 5 (Fig. 5E; Fig. S7E). The expression of the mesendoderm markers (*Eomes*, *Mesp1*, and *Gata6*) decreased significantly at day 3 in inhibitor-treated *Dlc1*-knockout EBs compared with the control group, while the ectoderm marker (*Pax6*) expression returned to normal (Fig. 5F). Moreover, the cardiac mesoderm markers (*Hand1* and *Tbx20*) decreased after treatment with the inhibitor; however, the difference was not significant at day 5.

Collectively, our findings indicate that *Dlc1* promotes mesoderm specification by activating the canonical Wnt signaling, thereby enhancing cardiac progenitor differentiation.

### *Dlc1* deletion activates the canonical Wnt pathway by modulating β-catenin

**Figure 6:**
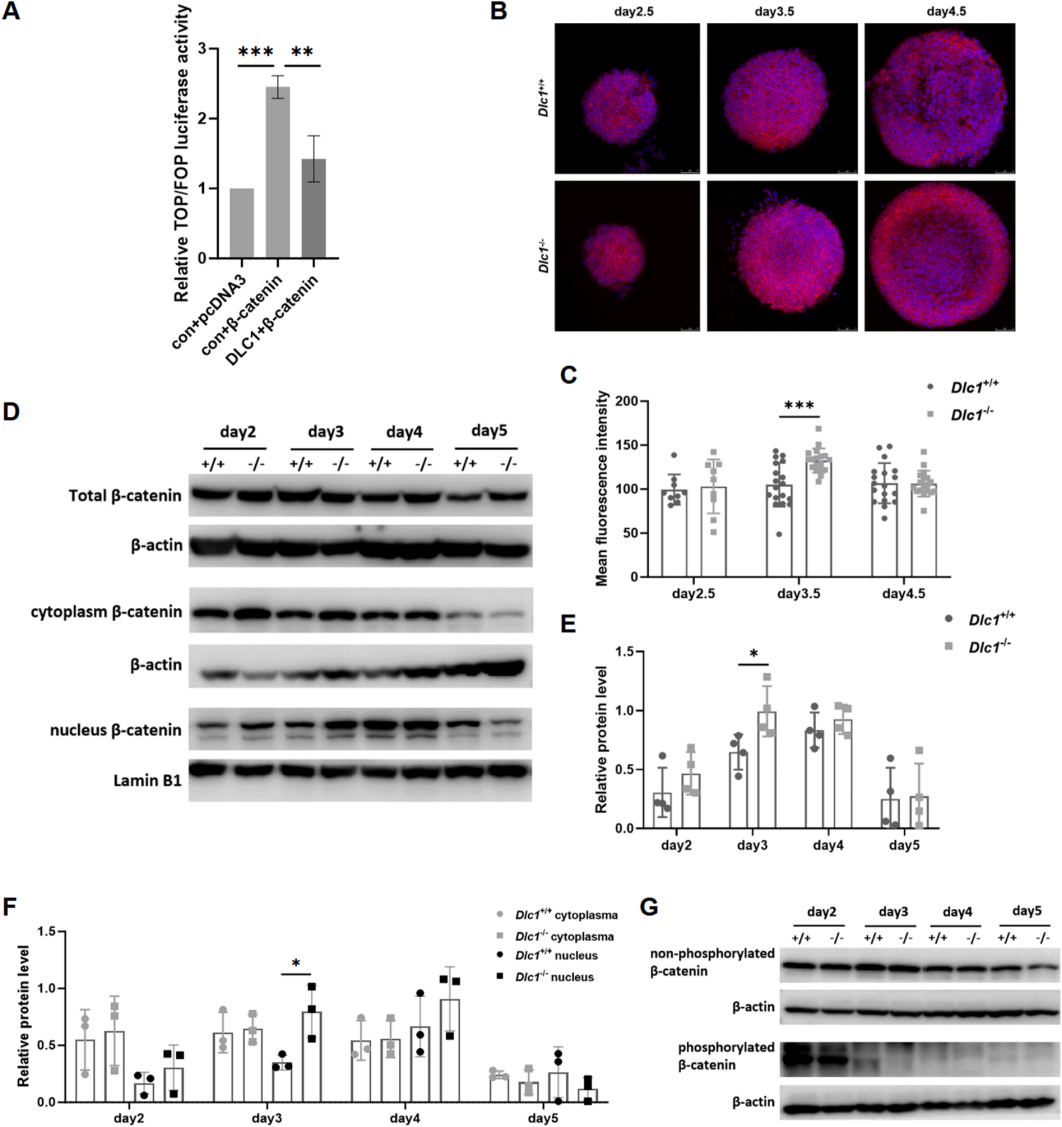
*Dlc1* knockout activates the canonical Wnt pathway via modulating β-catenin. **(A)** Luciferase activity of the TOP/FOP-flash reporter system regulated by blank vector, β-catenin, and β-catenin plus Dlc1. *n* = 4. **(B)** Representative immunofluorescent staining images of embryonic bodies (EBs) during differentiation. Red, β-catenin; Blue, DAPI. Scale bar, 200 μm. **(C)** Quantification of fluorescence intensity for β-catenin. *n* ≥ 9. **(D)** Representative Western blotting results for β-catenin expression at different subcellular locations during mESC differentiation. **(E)** Quantification of Western blotting results for total β-catenin. *n* ≥ 3. **(F)** Quantification of Western blotting results for cytoplasm and nucleus β-catenin. *n* = 3. **(G)** Western blotting results for phosphorylated and non-phosphorylated β-catenin. Data were shown as mean ± standard deviation; ∗*P* < 0.05 and ∗∗*P* < 0.01.

To confirm the role of *Dlc1* in regulating the canonical Wnt pathway, we used the TOP/FOP-flash reporter system to evaluate its function in the presence of β-catenin. Luciferase reporter gene analysis demonstrated that *Dlc1* overexpression down-regulated Wnt/β-catenin signaling activity compared with the control group (Fig. 6A).

To further elucidate the mechanism by which *Dlc1* activates the canonical Wnt pathway, we evaluated the expression of β-catenin during cardiac progenitor differentiation. We performed immunofluorescence staining at day 2.5–4.5 and found that β-catenin was mainly expressed on the cell membrane, with some distributed in the cytoplasm and nucleus (Fig. 6B). The expression of β-catenin increased at day 3.5 in *Dlc1*-knockout EBs (Fig. 6C), confirmed by Western blotting (Fig. 6D and E). Considering that β-catenin needs to translocate to the nucleus before affecting downstream transcription, we evaluated the expression of β-catenin in the cytoplasm and nucleus separately.^20^ β-catenin expression did not change in the cytoplasm; however, it increased in the nucleus at day 3 (Fig. 6D–F). Hence, during mesodermal differentiation, increased β-catenin expression leads to increased translocation to the nucleus and the up-regulation of downstream gene expression.

According to previous research, β-catenin needs to form a complex and be phosphorylated to degrade when Wnt signaling is in the off-state.^20^ We measured the hypo-phosphorylation and phosphorylation levels of β-catenin to explore how it accumulated in mesodermal progenitors. In *Dlc1*-knockout EBs, phosphorylation of β-catenin decreased at day 3, reducing the degradation of β-catenin protein during this stage (Fig. 6G). Therefore, *Dlc1* deletion activates the canonical Wnt pathway by reducing the degradation and promoting the translocation of β-catenin.

## Discussion

There are three main sources of cardiac cell precursors known to contribute to myocyte and non-myocyte cell lineages of the heart, namely, cardiac mesoderm cells, proepicardium, and cardiac neural crest cells.^2^ The cardiac mesoderm is the major source of the myocardium, and it also contributes to the endocardium and conduction system. In this study, we performed the *in vitro* cardiomyocyte differentiation using wild-type and *Dlc1*-knockout mESC to investigate the role of *Dlc1* in cardiac lineage specification. Our results revealed that *Dlc1* suppression improved cardiac mesoderm specification but impaired the subsequent differentiation of myocardial cells. Based on our RNA-sequencing analysis, we discovered that promoting mesoderm specification through activating the canonical Wnt signaling may be responsible for the enhanced differentiation of cardiac progenitor cells. In addition, the disrupted differentiation induced by *Dlc1* deficiency could be rescued by the Wnt/β-catenin inhibitor. We further validated the modulation of β-catenin and found that *Dlc1* deletion reduced its degradation and promoted its translocation.

Our results demonstrated that *Dlc1* deletion did not affect the self-renewal and expression of pluripotent factors (*e.g.*, Pou5f1, Nanog, and Klf2) in mESC,^21^^,^^22^ indicating that this gene may not be involved in mESC proliferation and pluripotency. However, during *in vitro* cardiomyocyte differentiation, *Dlc1* deletion disrupted the germ-layer and cardiac lineage commitment following the exit from pluripotency. The cardiac lineage is originally derived from the lateral plate mesoderm, which consists of cells migrating from the primitive streak to the anterior lateral regions of the embryo.^23^ The mesoderm lineage commitment is governed by the network of lineage-determining transcription factors and crosstalk between the developmental cues emanating from different germinal layers.^24^ During this process, activating the key T-box transcription factor, eomesodermin (*Eomes*), subsequently activates *Mesp1* and induces mESC differentiation into mesendodermal progenitors.^25^^,^^26^ *Mesp1* expression is transient during mesoderm formation, contributing to about 70% of cardiac cells, according to previous lineage tracing analysis.^26^ Our study found that mesoderm lineage specification was significantly promoted in *Dlc1*-knockout EBs and that the expression of key mesodermal transcription factors, such as *Eomes* and *Mesp1*, was up-regulated. Additionally, Shh signaling ligands expressed by the neighboring endoderm regulate mesodermal cardiopulmonary progenitor differentiation, while the deficiency of certain endodermal transcription factors, such as *Foxa2*, *Gata4*, and *Gata6*, impairs endoderm lineage and subsequent cardiac myocyte differentiation.^26^, ^27^, ^28^ We identified a promotion of endoderm lineage specification in *Dlc1*-knockout EBs compared with the control group and a significant increase of endodermal markers (*e.g.*, *Foxa2* and *Gata6*), which may also benefit mesodermal progenitor differentiation.

The cell fate specification of mesodermal cells to cardiac progenitors, corresponding to the cardiac crescent originating from the lateral plate mesoderm during gastrulation, is the key step controlled by complex signals during cardiomyocyte differentiation. Previous research demonstrated that promoting mesoderm differentiation using the CHIR (selective GSK-3 inhibitor) or Linc1405/Eomes complex promoted differentiation to the cardiac lineage, indicating that promoting the cardiac mesoderm lineage specification in *Dlc1*-knockout EBs may be due to increased mesodermal progenitor differentiation.^29^^,^^30^ However, other mechanisms may also exist. Embryonic heart development begins on both sides of the embryo, with cardiac progenitor cells from the first heart field (FHF) migrating to form the heart tube, and the other set of progenitors from the second heart field (SHF) patterning into the heart tube for elongation.^31^^,^^32^ Cardiac progenitors of the FHF and SHF, arising from mesodermal cells expressing *Mesp1,* are often identified by the positive expression of *Nkx2.5* and *Isl1*, separately.^26^^,^^33^ In *Dlc1*-knockout EBs, we observed an increase in *Isl1* expression at day 4, while the expression of *Nkx2.5* did not change during differentiation. Subsequently, the expression of the additional cardiac progenitor marker (*Hand1*) and several cardiac mesodermal transcription factors (*e.g.*, *Bmp4*, *Tbx2*, *Smarcd3*, and *Hand2*) was significantly up-regulated at day 5. *Bmp4* and *Smarcd3* are expressed in the SHF to control its morphogenesis, while *Hand1* and *Hand2* are proven to be related to FHF development.^34^, ^35^, ^36^ Although Owen et al discovered that *Isl1* was transiently expressed in the FHF, its role in FHF development was limited.^37^ Notably, according to GO and KEGG enrichment, the TGF-β signaling pathway was enriched at day 5 (Fig. S7A), which was confirmed to be involved in heart morphogenesis and the FHF/SHF lineage arising, suggesting that these pathways may play a role in *Dlc1* deletion-induced cardiac progenitor differentiation disorder.^38^^,^^39^ Although the specification of cardiac progenitors was promoted in *Dlc1*-knockout EBs, we found that certain cardiomyocyte markers, such as *Tnnt2* and *Myh6*, were down-regulated since day 8. In previous research, the markedly expanded SHF induced by stabilized β-catenin expression resulted in abnormal heart morphogenesis and cardiomyocyte specification.^40^ We believe that *Dlc1* deficiency promoted cardiac progenitor differentiation and accumulation; however, the subsequent cardiomyocyte lineage specification was blocked.

DLC1 protein is involved in regulating multiple development-related signaling pathways, such as EGFR/Akt/NF-κB, Wnt/β-catenin, and RhoA/ROCK.^41^ Among these, WNT signaling is instrumental in distinct steps during cardiogenesis. Its activation during mesoderm formation and inhibition during late cardiac progenitor specification are essential for myocardial cell differentiation.^20^ Notably, *Dlc1* expression was opposite to WNT signaling, with *Dcl1* expression being lower at the mesodermal stage and subsequently elevated. During germ layer induction, EOMES mostly functions by the cooperative interplay with the canonical WNT pathway, while Oct4 and the canonical WNT pathway regulate *Mesp1* expression.^42^^,^^43^ We observed the activation of the canonical Wnt pathway in *Dlc1*-knockout EBs at day 3, which resulted in promoting the mesodermal lineage specification. Moreover, Yibing et al demonstrated that activating the Wnt/β-catenin signaling in *Isl1*-positive progenitors of the SHF led to their accumulation and inhibition of further differentiation.^40^ In *Dlc1*-knockout EBs, we identified increased *Isl1* expression at day 4 when the canonical Wnt signaling was activated, indicating that the accumulation of cardiac progenitors may result from Wnt signaling regulation. Notably, in a Wnt pathway-related study, the mesoderm markers (*MIXL1*, *EOMES*, and *T*) were strongly induced by activin in *YAP*-knockout hESCs than in the wild-type, followed by subsequent elevation of cardiac mesoderm markers (*HAND1*, *GATA4*, and *Isl1*).^44^ Although Wnt signaling was down-regulated since day 5, further differentiation of cardiac progenitors to cardiomyocytes was still impaired in *Dlc1*-knockout EBs.

The canonical Wnt pathway is activated by the interaction between Wnt ligands and the receptor complex of Fzd and LRP5/6. Subsequently, β-catenin translocates to the nucleus and affects downstream transcription.^20^ DLC1 was found to regulate Wnt/β-catenin signaling by affecting β-catenin expression, thereby inhibiting cell growth and migration in colon cancer cells.^45^^,^^46^ We observed that the expression of the canonical Wnt pathway-related genes, such as secreted Wnt ligands, Fzd receptors, and β-catenin, was up-regulated in *Dlc1*-knockout EBs at day 3. Simultaneously, we found that more β-catenin was translocated to the nucleus to influence downstream transcription. For degradation, β-catenin was shown to first phosphorylate in a destruction complex comprising Axin, glycogen synthase kinase 3 beta (Gsk-3β), casein kinase 1 (Ck1), and adenomatous polyposis coli (Apc). Chunyi et al found that DLC1 overexpression increased the expression of GSK-3β in colon cancer cells, while it decreased the expression of β-catenin and its downstream target gene c-myc.^46^ We observed that β-catenin phosphorylation decreased at day 3; therefore, *Dlc1* influenced β-catenin protein accumulation and translocation at this stage.

Collectively, we found that the expression of *Dlc1* dynamically changed during the differentiation of mESC into cardiomyocytes. *Dlc1* deficiency promoted mesoderm lineage specification by activating canonical Wnt signaling, contributing to the accumulation of cardiac progenitors and ultimately blocking further differentiation into cardiomyocytes. Our results provided a new candidate gene related to cardiomyocyte differentiation and uncovered the possible mechanisms underlying the process, aiming to help elucidate the pathogenic genetic background of cardiac disease.

## CRediT authorship contribution statement

**Wei Wei:** Writing – review & editing, Visualization, Investigation, Writing – original draft, Methodology, Data curation. **Xuechao Jiang:** Investigation, Methodology, Data curation. **Wenxiang Shi:** Investigation, Software. **Junmei Zhou:** Supervision, Conceptualization, Project administration. **Rang Xu:** Supervision, Funding acquisition, Project administration, Conceptualization.

## Ethics declaration

This study was approved by the Local Ethics Committees of Xinhua Hospital (Shanghai, China) (No. XHEC-F-2023-019). All experiments on live vertebrates were performed in accordance with the institutional guidelines and regulations.

## Funding

This work was supported by the 10.13039/501100001809National Natural Science Foundation of China (No. 81970264).

## Conflict of interests

No conflict of interests exists in the submission of this manuscript, and the manuscript is approved by all authors for publication.
