# Supplementary material for: Dlc1 deletion activates the canonical Wnt pathway at an early stage and affects subsequent cardiac differentiation in mouse embryonic stem cells
Source: Genes Dis. 2025 Sep 9;13(6):101844. doi: 10.1016/j.gendis.2025.101844 (PMC13474022; doi:10.1016/j.gendis.2025.101844)
Supplement: Supplementary file 1 [file mmc1.docx]

**Supplementary Figure1.** Expression levels of DLC1 in human and mouse embryonic hearts. A. Expression pattern of CS11 to CS15 human embryonic hearts according to the gene chip expression profile . B. Expression pattern of E10.5 to E18.5 mouse embryonic hearts according to the published datasets (GSE1479). *n*=3. Data were shown as mean±SD; ***p*<0.01.

**Supplementary Figure2.** Verification of Dlc1 knockout mESC cell lines. A~C Sequencing chromatography of Dlc1 knockout cell lines. D. Verification of Dlc1 knockout mouse ES cell lines via detecting protein level.

**Supplementary Figure3.** *Dlc1* knockout did not affect the major biological activities of mESC. A. The proliferation rate of WT and *Dlc1*-KO EBs at day0 detected by flow cytometry. *n*=3. B. The apoptosis rate of WT and *Dlc1*-KO EBs at day0 detected by flow cytometry. *n*=3. C. Real-time PCR analysis of *Oct4* expression level at day0. *n*=6. Data were shown as mean±SD; ns not significant. D. GO analysis highlights pathways at day0. E. The heatmap represents pluripotency-related genes in the control EBs vs *Dlc1*-KO EBs at day0.

**Supplementary Figure4.** Expression levels of Nkx2.5 during mESC cardiomyocytes differentiation process. A. Expression pattern of Nkx2.5 in WT and *Dlc1*-KO Nkx2.5-emGFP mESC cell line detected by flow cytometry. *n*=4. B. Real-time PCR analysis of Nkx2.5 expression level during mESC cardiomyocytes differentiation. *n*=3. Data were shown as mean±SD; **p*<0.05, ***p*<0.01.

**Supplementary Figure5.** *Dlc1* knockout affects the efficiency of cardiomyocyte differentiation. A, Quantification of the percentage of beating focal in WT and two *Dlc1*-KO cell lines during differentiation. *n*=2. B, Expression pattern of Nkx2.5 in WT and two *Dlc1*-KO Nkx2.5-emGFP mESC cell lines detected by flow cytometry. *n*=2. C, Real-time PCR analysis of *Mesp1* expression of three *Dlc1*-KO lines. D, E, H, I, Real-time PCR analysis of *Nkx2.5*, *Isl1*, *Tnnt2* and *Myh6* expression of *Dlc1*-KO line 1 during mESC differentiation. F, G, J, K, Real-time PCR analysis of *Nkx2.5*, *Isl1*, *Tnnt2* and *Myh6* expression of *Dlc1*-KO line 3 during mESC differentiation. *n*=3. Data were shown as mean±SD; *P<0.05, **P<0.01, ***P<0.001.

**Supplementary Figure6.** Cardiac-related differentially expression genes during mESC cardiomyocytes differentiation process. A, B The volcano plot of differentially expression genes related to mesoderm or endoderm development according to GO terms at day3. C, D The volcano plot of differentially expression genes related to heart development according to GO terms at day5 or day9. Fold Change>2 or <-2, *P*.value<0.05.

**Supplementary Figure7.** Pathway analysis during mESC cardiomyocytes differentiation process. A, B KEGG analysis highlights pathways at day5 and day9. C, D GSEA analysis reveals the differentially expressed genes related to Wnt signaling pathways at day5 and day9. NES, normalized enrichment score. E. The proliferation rate of WT and *Dlc1*-KO EBs under indicating treatment at day3 and day5 detected by flow cytometry. *n*=3. Data were shown as mean±SD; ns, not significant; **p*<0.05.

**Supplementary Table1.** Primers for gRNA

| **Primer** | **Sequence** |
| --- | --- |
| gRNA 1-F | 5’-CACCGAGCATCTATGATAACGTAC-3’ |
| gRNA 1-R | 5’-AAACGTACGTTATCATAGATGCTC-3’ |
| gRNA 2-F | 5’-CACCGCGGAGTTGAGGCTCGGCCGG-3’ |
| gRNA 2-R | 5’-AAACCCGGCCGAGCCTCAACTCCGC-3’ |
| gRNA 3-F | 5’-CACCGACTGTGAACGTGCAGCGCTC-3’ |
| gRNA 3-R | 5’-AAACGAGCGCTGCACGTTCACAGTC-3’ |

**Supplementary Table2.** Primers for Verification and Real-time PCR

| **Primer** | **Sequence** |
| --- | --- |
| gRNA 1/2-F | 5’-GGTACGGAAAAGGAGCGTGT-3’ |
| gRNA 1/2-R | 5’-TTTTTGCCAGAAAGGTCCGC-3’ |
| gRNA 3-F | 5’-AGCAGGGCGTTTTACACTCA-3’ |
| gRNA 3-R | 5’-GGTCCAGACAGTGGTTACGG-3’ |
| *Oct4*-F | 5’-GCTCACCCTGGGCGTTCTCTTTGGA-3’ |
| *Oct4*-R | 5’-GGTTCTCATTGTTGTCGGCTTCCTCCAC-3’ |
| *Tnnt2*-F | 5’-CCGAGCAGCAGCGTATTCGCAATGAG-3’ |
| *Tnnt2*-R | 5’-AGCCTTCCTCCTGTTCTCCTCCTCCTC-3’ |
| *Myh6*-F | 5’-GATGGCACAGAAGATGCTGA-3’ |
| *Myh6*-R | 5’-CTGCCCCTTGGTGACATACT-3’ |
| *Isl1*-F | 5’-ACCCTCTCAGTCCCTTGCATCCA-3’ |
| *Isl1*-R | 5’-TCGTTGAGCACAGTCCGCACTC-3’ |
| *Nkx2.5*-F | 5’-CCCAAGTGCTCTCCTGCTTTCCCA-3’ |
| *Nkx2.5*-R | 5’-GGCTTTGTCCAGCTCCACTGCCTTCT-3’ |
| *Mesp1*-F | 5’-TGTACGCAGAAACAGCATCC-3’ |
| *Mesp1*-R | 5’-TTGTCCCCTCCACTCTTCAG-3’ |
| *Eomes*-F | 5’-GCGCATGTTTCCTTTCTTGAG-3’ |
| *Eomes*-R | 5’-GGTCGGCCAGAACCACTTC-3’ |
| *Gata6*-F | 5’-TTGCTCCGGTAACAGCAGTG-3’ |
| *Gata6*-R | 5’-GTGGTCGCTTGTGTAGAAGGA-3’ |
| *Gata4*-F | 5’-GCCTCTATCACAAGATGAACGGC-3’ |
| *Gata4*-R | 5’-TACAGGCTCACCCTCGGCATTA-3’ |
| *Pax6*-F | 5’-TGGCAAACAACCTGCCTATG-3’ |
| *Pax6*-R | 5’-TGCACGAGTATGAGGAGGTCT-3’ |
| *Nes*-F | 5’-CCCTGAAGTCGAGGAGCTG-3’ |
| *Nes*-R | 5’-CTGCTGCACCTCTAAGCGA-3’ |
| *Tbx20*-F | 5’-TATTCAGCATACTCCTAC-3’ |
| *Tbx20*-R | 5’-GTTAGTCTTGTCAATACG-3’ |
| *Hand1*-F | 5’-GGAGTTGCCTCAGCAGCCCG-3’ |
| *Hand1*-R | 5’-CCGTAGCCGCTGCGTCCTTT-3’ |
| *Gapdh*-F | 5’-TTCCGTGTTCCTACCCCCAATGTGT-3’ |
| *Gapdh-*R | 5’-GGAGTTGCTGTTGAAGTCGCAGGAG-3’ |

**Supplementary Table 3.** The bioinformatics information of *DCL1*^-/-^ cell lines

| **Cell line** | **Mutation site**  **(NM_001194940.2)** | **Amino acid change**  **(NP_001181869.1)** | **Exonic Function** | |
| --- | --- | --- | --- | --- |
| *Dlc1*^-/-^ -1 | c.[3301_3307del];[3301_3307del] | p.[ Arg1101Aspfs*41 Arg1101Aspfs*41] | | frameshift deletion |
| *Dlc1*^-/-^ -2 | c.[3290_3333del];[3290_3333del] | p.[Val1097Alafs*8];[Val1097Alafs*8] | frameshift deletion | |
| *Dlc1*^-/-^ -3 | c.[2496_2695del;3067_3076del];  [2496_2695del;3067_3076del] | p.[Thr833Phefs*16];[Thr833Phefs*16] | frameshift deletion | |
